# 1-[(Cyclo­hexyl­idene)amino]-3-(prop-2-en-1-yl)thio­urea

**DOI:** 10.1107/S1600536814014834

**Published:** 2014-06-25

**Authors:** Shaaban K. Mohamed, Joel T. Mague, Mehmet Akkurt, Alaa A. Hassan, Mustafa R. Albayati

**Affiliations:** a Chemistry and Environmental Division, Manchester Metropolitan University, Manchester M1 5GD, England; b Chemistry Department, Faculty of Science, Minia University, 61519 El-Minia, Egypt; c Department of Chemistry, Tulane University, New Orleans, LA 70118, USA; d Department of Physics, Faculty of Sciences, Erciyes University, 38039 Kayseri, Turkey; e Kirkuk University, College of Science, Department of Chemistry, Kirkuk, Iraq

**Keywords:** crystal structure

## Abstract

The asymmetric unit of the title compound, C_10_H_17_N_3_S, consists of three symmetry-independent mol­ecules with distinctly different conformations, as indicated for example by the C—N—C—C torsion angles of −155.9 (3), 89.9 (3) and 81.1 (4)° along the bond between thio­urea and allyl units. In the crystal, mol­ecules are connected *via* N—H⋯N and N—H⋯S hydrogen bonds into chains extending along [110] that are further associated through C—H⋯N inter­actions into layers parallel to (001). The allyl group in one of the independent mol­ecules is disordered over two sets of sites with an occupancy ratio of 0.853 (6):0.147 (6).

## Related literature   

For the use of thio­semicarbazides as inter­mediates in the synthesis of different heterocyclic compounds, see: Mague *et al.* (2014 ▶); Mohamed *et al.* (2014 ▶); Akkurt *et al.* (2014*a* ▶). For the bioactivity of thio­semicarbazones, see: Bahat *et al.* (2006 ▶); Qandil *et al.* (2006 ▶); Singh *et al.* (2001 ▶; Kalyoncuoğlu *et al.* (1992 ▶) Bahadur & Goel (1976 ▶). For the synthesis of the title compound, see: Akkurt *et al.* (2014*b* ▶).
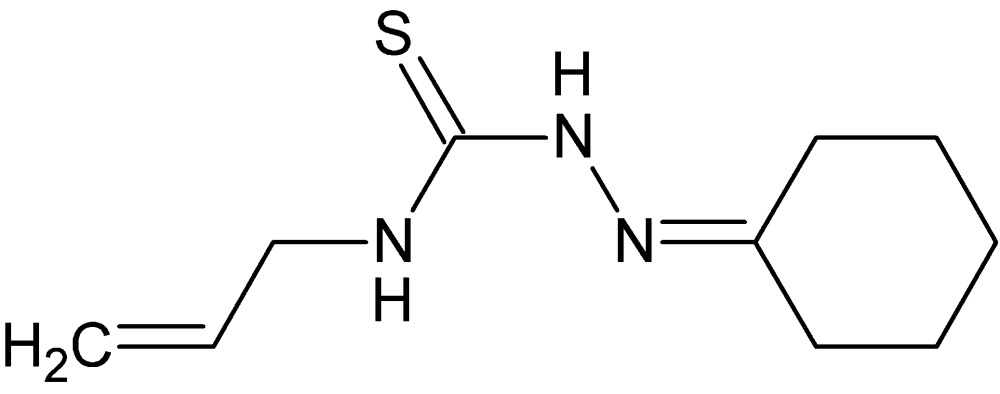


## Experimental   

### 

#### Crystal data  

C_10_H_17_N_3_S
*M* *_r_* = 211.32
Triclinic, 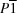

*a* = 8.4772 (15) Å
*b* = 11.180 (2) Å
*c* = 19.766 (4) Å
α = 77.864 (3)°
β = 82.777 (3)°
γ = 74.879 (3)°
*V* = 1763.0 (5) Å^3^
*Z* = 6
Mo *K*α radiation
μ = 0.24 mm^−1^
*T* = 150 K
0.26 × 0.15 × 0.10 mm

#### Data collection  

Bruker SMART APEX CCD diffractometer
Absorption correction: multi-scan (*SADABS*; Bruker, 2013 ▶) *T*_min_ = 0.65, *T*_max_ = 0.98
31443 measured reflections
8701 independent reflections
5402 reflections with *I* > 2σ(*I*)
*R*_int_ = 0.080

#### Refinement  

*R*[*F* ^2^ > 2σ(*F* ^2^)] = 0.072
*wR*(*F* ^2^) = 0.217
*S* = 1.04
8701 reflections
383 parameters
2 restraints
H-atom parameters constrained
Δρ_max_ = 0.70 e Å^−3^
Δρ_min_ = −0.60 e Å^−3^

### 

Data collection: *APEX2* (Bruker, 2013 ▶); cell refinement: *SAINT* (Bruker, 2013 ▶); data reduction: *SAINT*; program(s) used to solve structure: *SHELXT* (Sheldrick, 2008 ▶); program(s) used to refine structure: *SHELXL2014* (Sheldrick, 2008 ▶); molecular graphics: *DIAMOND* (Brandenburg & Putz, 2012 ▶); software used to prepare material for publication: *SHELXTL* (Sheldrick, 2008 ▶).

## Supplementary Material

Crystal structure: contains datablock(s) I, global. DOI: 10.1107/S1600536814014834/gk2613sup1.cif

Structure factors: contains datablock(s) I. DOI: 10.1107/S1600536814014834/gk2613Isup2.hkl

Supporting information file. DOI: 10.1107/S1600536814014834/gk2613Isup3.cml

CCDC reference: 1009841

Additional supporting information:  crystallographic information; 3D view; checkCIF report

## Figures and Tables

**Table 1:** Hydrogen-bond geometry (Å, °)

*D*—H⋯*A*	*D*—H	H⋯*A*	*D*⋯*A*	*D*—H⋯*A*
N8—H8⋯S1^i^	0.91	2.47	3.356 (2)	164
N1—H1*A*⋯N6	0.91	2.40	3.185 (3)	145
N2—H2*A*⋯S3^ii^	0.91	2.42	3.290 (2)	161
C10—H10*B*⋯N9^iii^	0.99	2.67	3.590 (4)	155
N4—H4*A*⋯N3	0.91	2.15	2.979 (3)	152
N5—H5*A*⋯N9	0.91	2.24	3.153 (3)	176
C16—H16*B*⋯N9	0.99	2.42	3.410 (4)	177
N7—H7⋯S2	0.91	2.51	3.363 (2)	155
C26—H26*A*⋯N2^iv^	0.99	2.67	3.626 (4)	163
C26—H26*A*⋯N3^iv^	0.99	2.60	3.499 (4)	152

Symmetry codes: (i) 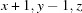; (ii) 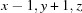; (iii) 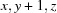; (iv) 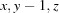.

**Fig. 1.**
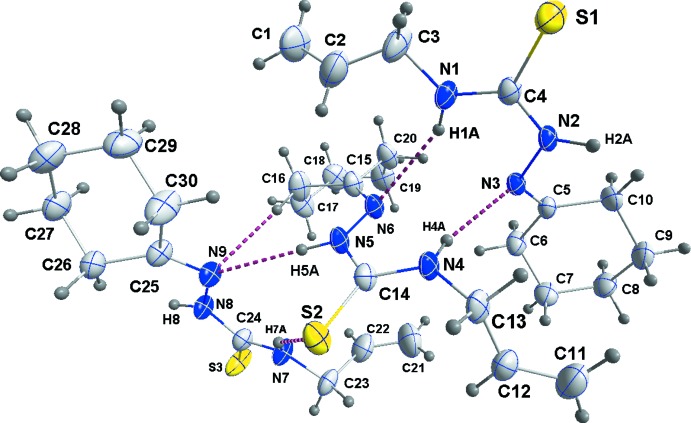
Asymmetric unit of the title compound with intermolecular hydrogen bonds shown as dotted lines. Ellipsoids are drawn at the 50% probability level. Only the major contributor to the disordered allyl group in the molecule 1 (with S1) is shown.

**Fig. 2.**
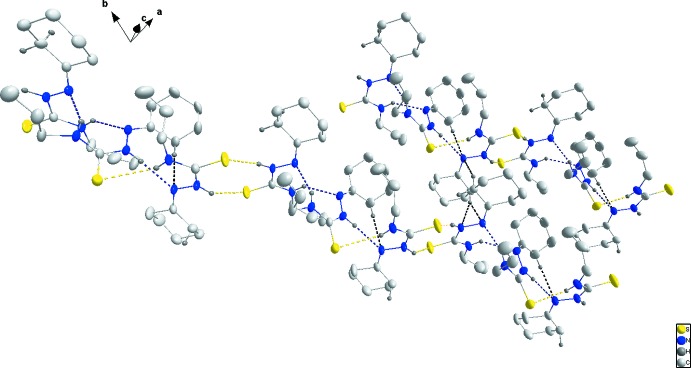
Packing viewed down the c axis showing intermolecular hydrogen bonding interactions as dotted lines (blue: N—H···N; yellow: N—H···S; black: C—H···N). For the sake of clarity, H atoms, not involved in hydrogen bonds, and minor component of the disordered group have been omitted.

## supplementary crystallographic information

### S1. Comment

It is well known that thiosemicarbazides are the key intermediates used in the synthesis of many heterocyclic compounds such as thiazolidinones (Mague *et al*., 2014), triazols (Mohamed *et al.*, 2014) and thiazoles (Akkurt *et al.*, 2014*a*). Thiosemicarbazone derivatives have displayed various pharmacological properties such as analgesic (Bahat *et al.*, 2006), anti-bacterial (Qandil *et al.*, 2006), anti-fungal, anti-tumoral (Singh *et al.*, 2001; Kalyoncuoğlu *et al.*, 1992) and anti-tubercular (Bahadur & Goel, 1976) activities. In view of these findings and as part of ongoing research in the domain of heterocyclic compounds of the 1,2,4-triazole class with expected biological activity we report the synthesis and crystal structure of the title compound.

The asymmetric unit consists of three independent molecules having distinctly different conformations as indicated by the torsion angles C4–N1–C3–C2 (-155.9 (3)°), C14–N4–C13–C12 (89.9 (3)°) and C24–N7–C23–C22 (81.1 (4)°) for the allyl group. These molecules are associated *via* N—H···N hydrogen bonds (Fig. 1 and Table 1).

The packing consists of layers parallel to (001) formed by molecules joined via N—H···N hydrogen bonds, N—H···S and C—H···N interactions (Fig. 2 and Table 1).

### S2. Experimental

The title compound was prepared according to our previously reported method (Akkurt *et al.*, 2014*b*). Colourless crystals suitable for X-ray diffraction were obtained by crystallization from ethanol (m.p. 438 K).

### S3. Refinement

H atoms attached to carbon were placed in calculated positions (C—H = 0.95 - 0.99 Å) while those attached to nitrogen (except H8 on N8) were placed in locations derived from a difference map and their coordinates adjusted to give N—H = 0.91 Å. All H atoms were included as riding contributions with isotropic displacement parameters 1.2 times those of the attached atoms. There wasn't a clear indication of the H8 atom on N8 from the difference map, despite seeing all of the others on N but it couldn't be seen any significant difference in the bond distances between the molecule with N8 and the other two so it was put it in the calculated position. In the molecule 1 (with S1), atoms C1, C2 and C3 of the allyl group are disordered over two sites, with refined occupancies of 0.853 (6) and 0.147 (6).

### Crystal data

**Table:**

C_10_H_17_N_3_S	*Z* = 6
*M**_r_* = 211.32	*F*(000) = 684
Triclinic, *P*1	*D*_x_ = 1.194 Mg m^−^^3^
*a* = 8.4772 (15) Å	Mo *K*α radiation, λ = 0.71073 Å
*b* = 11.180 (2) Å	Cell parameters from 9052 reflections
*c* = 19.766 (4) Å	θ = 2.4–28.2°
α = 77.864 (3)°	µ = 0.24 mm^−^^1^
β = 82.777 (3)°	*T* = 150 K
γ = 74.879 (3)°	Plate, colourless
*V* = 1763.0 (5) Å^3^	0.26 × 0.15 × 0.10 mm

### Data collection

**Table:**

Bruker SMART APEX CCD diffractometer	8701 independent reflections
Radiation source: fine-focus sealed tube	5402 reflections with *I* > 2σ(*I*)
Graphite monochromator	*R*_int_ = 0.080
Detector resolution: 8.3660 pixels mm^-1^	θ_max_ = 28.4°, θ_min_ = 1.9°
φ and ω scans	*h* = −11→11
Absorption correction: multi-scan (*SADABS*; Bruker, 2013)	*k* = −14→14
*T*_min_ = 0.65, *T*_max_ = 0.98	*l* = −26→26
31443 measured reflections	

### Refinement

**Table:**

Refinement on *F*^2^	2 restraints
Least-squares matrix: full	Hydrogen site location: mixed
*R*[*F*^2^ > 2σ(*F*^2^)] = 0.072	H-atom parameters constrained
*wR*(*F*^2^) = 0.217	*w* = 1/[σ^2^(*F*_o_^2^) + (0.111*P*)^2^ + 0.3498*P*] where *P* = (*F*_o_^2^ + 2*F*_c_^2^)/3
*S* = 1.04	(Δ/σ)_max_ = 0.001
8701 reflections	Δρ_max_ = 0.70 e Å^−^^3^
383 parameters	Δρ_min_ = −0.60 e Å^−^^3^

### Special details

**Table:**

Geometry. All e.s.d.'s (except the e.s.d. in the dihedral angle between two l.s. planes) are estimated using the full covariance matrix. The cell e.s.d.'s are taken into account individually in the estimation of e.s.d.'s in distances, angles and torsion angles; correlations between e.s.d.'s in cell parameters are only used when they are defined by crystal symmetry. An approximate (isotropic) treatment of cell e.s.d.'s is used for estimating e.s.d.'s involving l.s. planes.

### Fractional atomic coordinates and isotropic or equivalent isotropic displacement parameters (Å^2^)

**Table:**

	*x*	*y*	*z*	*U*_iso_*/*U*_eq_	Occ. (<1)
S1	−0.18746 (10)	1.02518 (8)	0.63133 (4)	0.0415 (2)	
N1	0.0924 (3)	0.8466 (2)	0.64330 (12)	0.0324 (6)	
H1A	0.1736	0.8045	0.6717	0.039*	
N2	0.0116 (3)	0.9571 (2)	0.73177 (11)	0.0260 (5)	
H2A	−0.0679	1.0047	0.7571	0.031*	
N3	0.1448 (3)	0.8754 (2)	0.76609 (11)	0.0258 (5)	
C1	0.2643 (5)	0.6623 (4)	0.5143 (2)	0.0625 (11)	0.853 (6)
H1B	0.2847	0.7253	0.4761	0.075*	0.853 (6)
H1C	0.3154	0.5760	0.5138	0.075*	0.853 (6)
C2	0.1662 (5)	0.6941 (4)	0.5672 (2)	0.0444 (11)	0.853 (6)
H2	0.1499	0.6275	0.6040	0.053*	0.853 (6)
C3	0.0807 (4)	0.8189 (4)	0.57564 (18)	0.0550 (11)	0.853 (6)
H3A	−0.0363	0.8312	0.5684	0.066*	0.853 (6)
H3B	0.1249	0.8799	0.5394	0.066*	0.853 (6)
C1A	0.2643 (5)	0.6623 (4)	0.5143 (2)	0.0625 (11)	0.147 (6)
H1A1	0.1897	0.6095	0.5234	0.075*	0.147 (6)
H1A2	0.3662	0.6377	0.4886	0.075*	0.147 (6)
C2A	0.2270 (15)	0.7682 (12)	0.5368 (10)	0.0444 (11)	0.147 (6)
H2A1	0.3062	0.8172	0.5261	0.053*	0.147 (6)
C3A	0.0807 (4)	0.8189 (4)	0.57564 (18)	0.0550 (11)	0.147 (6)
H3A1	0.0097	0.7591	0.5823	0.066*	0.147 (6)
H3A2	0.0232	0.8980	0.5467	0.066*	0.147 (6)
C4	−0.0192 (3)	0.9360 (3)	0.67085 (14)	0.0274 (6)	
C5	0.2028 (3)	0.9152 (3)	0.81122 (14)	0.0253 (6)	
C6	0.3500 (4)	0.8286 (3)	0.84516 (15)	0.0362 (7)	
H6A	0.4481	0.8611	0.8270	0.043*	
H6B	0.3678	0.7441	0.8333	0.043*	
C7	0.3291 (4)	0.8170 (3)	0.92401 (16)	0.0411 (8)	
H7A	0.2435	0.7708	0.9431	0.049*	
H7B	0.4331	0.7679	0.9441	0.049*	
C8	0.2810 (4)	0.9459 (3)	0.94482 (16)	0.0398 (8)	
H8A	0.3711	0.9890	0.9297	0.048*	
H8B	0.2628	0.9356	0.9960	0.048*	
C9	0.1254 (4)	1.0258 (3)	0.91177 (16)	0.0378 (7)	
H9A	0.0977	1.1100	0.9249	0.045*	
H9B	0.0336	0.9856	0.9297	0.045*	
C10	0.1460 (4)	1.0411 (3)	0.83286 (15)	0.0306 (6)	
H10A	0.0401	1.0865	0.8133	0.037*	
H10B	0.2268	1.0920	0.8143	0.037*	
S2	0.04448 (9)	0.38536 (7)	0.81703 (4)	0.0344 (2)	
N4	0.0610 (3)	0.6257 (2)	0.80237 (12)	0.0295 (5)	
H4A	0.1215	0.6842	0.7892	0.035*	
N5	0.2582 (3)	0.5009 (2)	0.74072 (12)	0.0293 (5)	
H5A	0.3012	0.4213	0.7326	0.035*	
N6	0.3309 (3)	0.6004 (2)	0.72156 (12)	0.0288 (5)	
C11	−0.0870 (5)	0.7195 (4)	0.96373 (19)	0.0531 (10)	
H11A	−0.1571	0.7992	0.9473	0.064*	
H11B	−0.0530	0.7003	1.0096	0.064*	
C12	−0.0371 (4)	0.6360 (3)	0.92302 (16)	0.0405 (8)	
H12	0.0329	0.5573	0.9411	0.049*	
C13	−0.0823 (3)	0.6559 (3)	0.85079 (15)	0.0341 (7)	
H13A	−0.1575	0.6023	0.8489	0.041*	
H13B	−0.1414	0.7450	0.8364	0.041*	
C14	0.1222 (3)	0.5116 (3)	0.78668 (14)	0.0266 (6)	
C15	0.4660 (4)	0.5871 (3)	0.68289 (16)	0.0341 (7)	
C16	0.5607 (4)	0.4732 (3)	0.65407 (19)	0.0447 (8)	
H16A	0.5588	0.4914	0.6029	0.054*	
H16B	0.5088	0.4020	0.6726	0.054*	
C17	0.7356 (4)	0.4370 (3)	0.6734 (2)	0.0528 (10)	
H17A	0.7992	0.3682	0.6494	0.063*	
H17B	0.7381	0.4050	0.7240	0.063*	
C18	0.8153 (4)	0.5470 (3)	0.6541 (2)	0.0502 (9)	
H18A	0.8249	0.5727	0.6030	0.060*	
H18B	0.9271	0.5206	0.6704	0.060*	
C19	0.7170 (4)	0.6586 (4)	0.6860 (2)	0.0511 (9)	
H19A	0.7688	0.7305	0.6704	0.061*	
H19B	0.7168	0.6358	0.7372	0.061*	
C20	0.5420 (4)	0.6971 (3)	0.66504 (19)	0.0424 (8)	
H20A	0.4773	0.7651	0.6894	0.051*	
H20B	0.5413	0.7300	0.6145	0.051*	
S3	0.72249 (10)	0.06142 (9)	0.84757 (4)	0.0438 (2)	
N7	0.4364 (3)	0.2322 (2)	0.84226 (12)	0.0302 (5)	
H7	0.3393	0.2647	0.8224	0.036*	
N8	0.5182 (3)	0.1342 (2)	0.74897 (12)	0.0280 (5)	
H8	0.6012	0.0897	0.7236	0.034*	
N9	0.3906 (3)	0.2225 (2)	0.71413 (12)	0.0281 (5)	
C21	0.5004 (6)	0.4744 (4)	0.9126 (2)	0.0694 (12)	
H21A	0.3913	0.4994	0.9320	0.083*	
H21B	0.5701	0.5309	0.9045	0.083*	
C22	0.5541 (5)	0.3631 (4)	0.89692 (18)	0.0481 (9)	
H22	0.6638	0.3413	0.8775	0.058*	
C23	0.4558 (4)	0.2665 (3)	0.90719 (15)	0.0342 (7)	
H23A	0.5105	0.1901	0.9390	0.041*	
H23B	0.3462	0.3001	0.9293	0.041*	
C24	0.5503 (3)	0.1497 (3)	0.81134 (14)	0.0280 (6)	
C25	0.3286 (3)	0.1857 (3)	0.66890 (14)	0.0277 (6)	
C26	0.3739 (4)	0.0583 (3)	0.64931 (15)	0.0324 (6)	
H26A	0.2857	0.0143	0.6676	0.039*	
H26B	0.4756	0.0079	0.6709	0.039*	
C27	0.4000 (4)	0.0670 (3)	0.57133 (16)	0.0379 (7)	
H27A	0.4164	−0.0179	0.5602	0.046*	
H27B	0.5003	0.0971	0.5541	0.046*	
C28	0.2550 (4)	0.1561 (3)	0.53470 (16)	0.0429 (8)	
H28A	0.2784	0.1628	0.4839	0.051*	
H28B	0.1568	0.1217	0.5483	0.051*	
C29	0.2207 (5)	0.2855 (3)	0.55305 (17)	0.0488 (9)	
H29A	0.1226	0.3404	0.5305	0.059*	
H29B	0.3147	0.3234	0.5352	0.059*	
C30	0.1925 (4)	0.2789 (3)	0.63118 (17)	0.0453 (9)	
H30A	0.1831	0.3633	0.6418	0.054*	
H30B	0.0879	0.2548	0.6477	0.054*	

### Atomic displacement parameters (Å^2^)

**Table:**

	*U*^11^	*U*^22^	*U*^33^	*U*^12^	*U*^13^	*U*^23^
S1	0.0271 (4)	0.0547 (5)	0.0338 (4)	0.0140 (4)	−0.0074 (3)	−0.0156 (4)
N1	0.0211 (12)	0.0413 (14)	0.0319 (12)	0.0078 (10)	−0.0051 (9)	−0.0177 (11)
N2	0.0182 (11)	0.0256 (12)	0.0302 (11)	0.0064 (9)	−0.0024 (9)	−0.0106 (9)
N3	0.0206 (12)	0.0242 (12)	0.0287 (11)	0.0037 (9)	−0.0026 (9)	−0.0072 (9)
C1	0.062 (3)	0.060 (2)	0.056 (2)	0.016 (2)	0.0015 (19)	−0.031 (2)
C2	0.053 (3)	0.046 (2)	0.038 (2)	−0.0102 (19)	−0.0007 (18)	−0.0207 (18)
C3	0.041 (2)	0.075 (3)	0.0433 (19)	0.0215 (18)	−0.0136 (15)	−0.0359 (18)
C1A	0.062 (3)	0.060 (2)	0.056 (2)	0.016 (2)	0.0015 (19)	−0.031 (2)
C2A	0.053 (3)	0.046 (2)	0.038 (2)	−0.0102 (19)	−0.0007 (18)	−0.0207 (18)
C3A	0.041 (2)	0.075 (3)	0.0433 (19)	0.0215 (18)	−0.0136 (15)	−0.0359 (18)
C4	0.0198 (13)	0.0309 (15)	0.0289 (13)	0.0003 (11)	0.0007 (10)	−0.0090 (11)
C5	0.0213 (13)	0.0241 (14)	0.0280 (13)	−0.0007 (11)	0.0013 (10)	−0.0071 (11)
C6	0.0271 (16)	0.0392 (17)	0.0385 (16)	0.0069 (13)	−0.0087 (12)	−0.0141 (13)
C7	0.0398 (19)	0.0418 (19)	0.0378 (16)	0.0016 (15)	−0.0114 (14)	−0.0079 (14)
C8	0.0381 (18)	0.0473 (19)	0.0334 (15)	−0.0014 (15)	−0.0072 (13)	−0.0146 (14)
C9	0.0365 (17)	0.0382 (17)	0.0389 (16)	−0.0005 (14)	−0.0020 (13)	−0.0184 (14)
C10	0.0305 (16)	0.0259 (14)	0.0366 (15)	−0.0042 (12)	−0.0052 (12)	−0.0102 (12)
S2	0.0239 (4)	0.0279 (4)	0.0488 (5)	−0.0030 (3)	0.0033 (3)	−0.0092 (3)
N4	0.0225 (12)	0.0242 (12)	0.0375 (13)	0.0019 (9)	0.0072 (10)	−0.0116 (10)
N5	0.0221 (12)	0.0221 (11)	0.0413 (13)	−0.0003 (9)	0.0065 (10)	−0.0124 (10)
N6	0.0211 (12)	0.0240 (12)	0.0384 (13)	−0.0003 (9)	0.0048 (10)	−0.0098 (10)
C11	0.057 (2)	0.057 (2)	0.047 (2)	−0.0156 (19)	0.0145 (17)	−0.0212 (18)
C12	0.0359 (18)	0.0404 (18)	0.0391 (17)	−0.0022 (14)	0.0107 (13)	−0.0109 (14)
C13	0.0230 (15)	0.0309 (15)	0.0425 (17)	0.0039 (12)	0.0086 (12)	−0.0133 (13)
C14	0.0176 (13)	0.0249 (14)	0.0339 (14)	0.0030 (11)	−0.0018 (10)	−0.0084 (11)
C15	0.0241 (15)	0.0304 (16)	0.0447 (17)	0.0015 (12)	0.0011 (12)	−0.0124 (13)
C16	0.0281 (17)	0.0439 (19)	0.061 (2)	−0.0027 (14)	0.0117 (15)	−0.0234 (17)
C17	0.0316 (19)	0.040 (2)	0.076 (3)	0.0067 (15)	0.0092 (17)	−0.0147 (18)
C18	0.0176 (16)	0.057 (2)	0.069 (2)	0.0004 (15)	0.0019 (15)	−0.0107 (19)
C19	0.0313 (19)	0.052 (2)	0.072 (3)	−0.0131 (16)	0.0013 (17)	−0.0159 (19)
C20	0.0339 (18)	0.0333 (17)	0.057 (2)	−0.0068 (14)	0.0084 (15)	−0.0101 (15)
S3	0.0250 (4)	0.0626 (6)	0.0341 (4)	0.0172 (4)	−0.0075 (3)	−0.0194 (4)
N7	0.0190 (12)	0.0371 (14)	0.0328 (12)	0.0051 (10)	−0.0028 (9)	−0.0173 (11)
N8	0.0193 (11)	0.0289 (12)	0.0315 (12)	0.0086 (9)	−0.0049 (9)	−0.0124 (10)
N9	0.0199 (11)	0.0270 (12)	0.0332 (12)	0.0069 (9)	−0.0039 (9)	−0.0112 (10)
C21	0.083 (3)	0.052 (2)	0.083 (3)	−0.018 (2)	−0.013 (3)	−0.027 (2)
C22	0.044 (2)	0.059 (2)	0.0467 (19)	−0.0097 (18)	−0.0062 (15)	−0.0238 (17)
C23	0.0267 (15)	0.0422 (17)	0.0314 (14)	0.0046 (13)	−0.0012 (11)	−0.0186 (13)
C24	0.0206 (14)	0.0311 (15)	0.0300 (14)	−0.0005 (11)	0.0003 (10)	−0.0095 (12)
C25	0.0182 (13)	0.0331 (15)	0.0298 (13)	0.0023 (11)	−0.0017 (10)	−0.0120 (12)
C26	0.0300 (16)	0.0334 (16)	0.0355 (15)	−0.0057 (13)	−0.0043 (12)	−0.0118 (13)
C27	0.0349 (17)	0.0428 (18)	0.0378 (16)	−0.0033 (14)	−0.0038 (13)	−0.0180 (14)
C28	0.0364 (18)	0.061 (2)	0.0345 (16)	−0.0107 (16)	−0.0052 (13)	−0.0157 (15)
C29	0.048 (2)	0.049 (2)	0.0439 (19)	0.0028 (17)	−0.0214 (16)	−0.0037 (16)
C30	0.0345 (18)	0.048 (2)	0.0474 (18)	0.0163 (15)	−0.0186 (14)	−0.0185 (16)

### Geometric parameters (Å, º)

**Table:**

S1—C4	1.688 (3)	C15—C20	1.492 (4)
N1—C4	1.341 (3)	C15—C16	1.499 (4)
N1—C3	1.455 (4)	C16—C17	1.507 (5)
N1—H1A	0.9099	C16—H16A	0.9900
N2—C4	1.343 (3)	C16—H16B	0.9900
N2—N3	1.401 (3)	C17—C18	1.512 (5)
N2—H2A	0.9099	C17—H17A	0.9900
N3—C5	1.276 (3)	C17—H17B	0.9900
C1—C2	1.303 (5)	C18—C19	1.517 (5)
C1—H1B	0.9500	C18—H18A	0.9900
C1—H1C	0.9500	C18—H18B	0.9900
C2—C3	1.428 (5)	C19—C20	1.518 (5)
C2—H2	0.9500	C19—H19A	0.9900
C3—H3A	0.9900	C19—H19B	0.9900
C3—H3B	0.9900	C20—H20A	0.9900
C2A—H2A1	0.9500	C20—H20B	0.9900
C5—C10	1.498 (4)	S3—C24	1.683 (3)
C5—C6	1.501 (4)	N7—C24	1.334 (3)
C6—C7	1.528 (4)	N7—C23	1.454 (3)
C6—H6A	0.9900	N7—H7	0.9099
C6—H6B	0.9900	N8—C24	1.350 (3)
C7—C8	1.521 (4)	N8—N9	1.401 (3)
C7—H7A	0.9900	N8—H8	0.9100
C7—H7B	0.9900	N9—C25	1.277 (3)
C8—C9	1.524 (4)	C21—C22	1.297 (5)
C8—H8A	0.9900	C21—H21A	0.9500
C8—H8B	0.9900	C21—H21B	0.9500
C9—C10	1.526 (4)	C22—C23	1.496 (5)
C9—H9A	0.9900	C22—H22	0.9500
C9—H9B	0.9900	C23—H23A	0.9900
C10—H10A	0.9900	C23—H23B	0.9900
C10—H10B	0.9900	C25—C30	1.495 (4)
S2—C14	1.678 (3)	C25—C26	1.495 (4)
N4—C14	1.331 (3)	C26—C27	1.515 (4)
N4—C13	1.461 (3)	C26—H26A	0.9900
N4—H4A	0.9099	C26—H26B	0.9900
N5—C14	1.370 (3)	C27—C28	1.520 (4)
N5—N6	1.372 (3)	C27—H27A	0.9900
N5—H5A	0.9099	C27—H27B	0.9900
N6—C15	1.288 (4)	C28—C29	1.511 (5)
C11—C12	1.312 (4)	C28—H28A	0.9900
C11—H11A	0.9500	C28—H28B	0.9900
C11—H11B	0.9500	C29—C30	1.522 (5)
C12—C13	1.481 (4)	C29—H29A	0.9900
C12—H12	0.9500	C29—H29B	0.9900
C13—H13A	0.9900	C30—H30A	0.9900
C13—H13B	0.9900	C30—H30B	0.9900
			
C4—N1—C3	122.8 (2)	C15—C16—H16B	109.6
C4—N1—H1A	113.0	C17—C16—H16B	109.6
C3—N1—H1A	124.2	H16A—C16—H16B	108.1
C4—N2—N3	118.5 (2)	C16—C17—C18	112.0 (3)
C4—N2—H2A	120.6	C16—C17—H17A	109.2
N3—N2—H2A	117.8	C18—C17—H17A	109.2
C5—N3—N2	117.6 (2)	C16—C17—H17B	109.2
C2—C1—H1B	120.0	C18—C17—H17B	109.2
C2—C1—H1C	120.0	H17A—C17—H17B	107.9
H1B—C1—H1C	120.0	C17—C18—C19	111.5 (3)
C1—C2—C3	126.7 (4)	C17—C18—H18A	109.3
C1—C2—H2	116.7	C19—C18—H18A	109.3
C3—C2—H2	116.7	C17—C18—H18B	109.3
C2—C3—N1	113.9 (3)	C19—C18—H18B	109.3
C2—C3—H3A	108.8	H18A—C18—H18B	108.0
N1—C3—H3A	108.8	C18—C19—C20	110.6 (3)
C2—C3—H3B	108.8	C18—C19—H19A	109.5
N1—C3—H3B	108.8	C20—C19—H19A	109.5
H3A—C3—H3B	107.7	C18—C19—H19B	109.5
N1—C4—N2	116.7 (2)	C20—C19—H19B	109.5
N1—C4—S1	123.5 (2)	H19A—C19—H19B	108.1
N2—C4—S1	119.8 (2)	C15—C20—C19	110.4 (3)
N3—C5—C10	127.8 (2)	C15—C20—H20A	109.6
N3—C5—C6	116.8 (2)	C19—C20—H20A	109.6
C10—C5—C6	115.3 (2)	C15—C20—H20B	109.6
C5—C6—C7	111.6 (2)	C19—C20—H20B	109.6
C5—C6—H6A	109.3	H20A—C20—H20B	108.1
C7—C6—H6A	109.3	C24—N7—C23	123.7 (2)
C5—C6—H6B	109.3	C24—N7—H7	117.3
C7—C6—H6B	109.3	C23—N7—H7	118.9
H6A—C6—H6B	108.0	C24—N8—N9	118.8 (2)
C8—C7—C6	111.3 (3)	C24—N8—H8	117.8
C8—C7—H7A	109.4	N9—N8—H8	118.6
C6—C7—H7A	109.4	C25—N9—N8	116.6 (2)
C8—C7—H7B	109.4	C22—C21—H21A	120.0
C6—C7—H7B	109.4	C22—C21—H21B	120.0
H7A—C7—H7B	108.0	H21A—C21—H21B	120.0
C7—C8—C9	110.5 (3)	C21—C22—C23	124.7 (4)
C7—C8—H8A	109.6	C21—C22—H22	117.6
C9—C8—H8A	109.6	C23—C22—H22	117.6
C7—C8—H8B	109.6	N7—C23—C22	112.3 (3)
C9—C8—H8B	109.6	N7—C23—H23A	109.1
H8A—C8—H8B	108.1	C22—C23—H23A	109.1
C8—C9—C10	111.5 (3)	N7—C23—H23B	109.1
C8—C9—H9A	109.3	C22—C23—H23B	109.1
C10—C9—H9A	109.3	H23A—C23—H23B	107.9
C8—C9—H9B	109.3	N7—C24—N8	116.5 (2)
C10—C9—H9B	109.3	N7—C24—S3	123.7 (2)
H9A—C9—H9B	108.0	N8—C24—S3	119.7 (2)
C5—C10—C9	110.8 (2)	N9—C25—C30	117.2 (3)
C5—C10—H10A	109.5	N9—C25—C26	127.7 (2)
C9—C10—H10A	109.5	C30—C25—C26	115.1 (2)
C5—C10—H10B	109.5	C25—C26—C27	111.7 (2)
C9—C10—H10B	109.5	C25—C26—H26A	109.3
H10A—C10—H10B	108.1	C27—C26—H26A	109.3
C14—N4—C13	123.6 (3)	C25—C26—H26B	109.3
C14—N4—H4A	118.6	C27—C26—H26B	109.3
C13—N4—H4A	116.7	H26A—C26—H26B	107.9
C14—N5—N6	118.9 (2)	C26—C27—C28	111.6 (3)
C14—N5—H5A	113.8	C26—C27—H27A	109.3
N6—N5—H5A	126.3	C28—C27—H27A	109.3
C15—N6—N5	119.1 (2)	C26—C27—H27B	109.3
C12—C11—H11A	120.0	C28—C27—H27B	109.3
C12—C11—H11B	120.0	H27A—C27—H27B	108.0
H11A—C11—H11B	120.0	C29—C28—C27	111.0 (3)
C11—C12—C13	124.6 (3)	C29—C28—H28A	109.4
C11—C12—H12	117.7	C27—C28—H28A	109.4
C13—C12—H12	117.7	C29—C28—H28B	109.4
N4—C13—C12	112.1 (2)	C27—C28—H28B	109.4
N4—C13—H13A	109.2	H28A—C28—H28B	108.0
C12—C13—H13A	109.2	C28—C29—C30	111.3 (3)
N4—C13—H13B	109.2	C28—C29—H29A	109.4
C12—C13—H13B	109.2	C30—C29—H29A	109.4
H13A—C13—H13B	107.9	C28—C29—H29B	109.4
N4—C14—N5	115.3 (2)	C30—C29—H29B	109.4
N4—C14—S2	125.5 (2)	H29A—C29—H29B	108.0
N5—C14—S2	119.2 (2)	C25—C30—C29	111.8 (3)
N6—C15—C20	116.9 (3)	C25—C30—H30A	109.2
N6—C15—C16	128.3 (3)	C29—C30—H30A	109.2
C20—C15—C16	114.8 (3)	C25—C30—H30B	109.2
C15—C16—C17	110.5 (3)	C29—C30—H30B	109.2
C15—C16—H16A	109.6	H30A—C30—H30B	107.9
C17—C16—H16A	109.6		
			
C4—N2—N3—C5	159.5 (3)	N6—C15—C16—C17	−125.8 (4)
C1—C2—C3—N1	−133.2 (4)	C20—C15—C16—C17	52.9 (4)
C4—N1—C3—C2	−155.9 (3)	C15—C16—C17—C18	−52.3 (4)
C3—N1—C4—N2	−176.0 (3)	C16—C17—C18—C19	55.4 (4)
C3—N1—C4—S1	1.1 (5)	C17—C18—C19—C20	−56.1 (4)
N3—N2—C4—N1	−7.8 (4)	N6—C15—C20—C19	124.5 (3)
N3—N2—C4—S1	175.00 (19)	C16—C15—C20—C19	−54.3 (4)
N2—N3—C5—C10	0.0 (4)	C18—C19—C20—C15	54.5 (4)
N2—N3—C5—C6	−177.4 (2)	C24—N8—N9—C25	−157.6 (3)
N3—C5—C6—C7	−132.0 (3)	C24—N7—C23—C22	81.1 (4)
C10—C5—C6—C7	50.4 (4)	C21—C22—C23—N7	118.9 (4)
C5—C6—C7—C8	−52.1 (4)	C23—N7—C24—N8	−175.8 (3)
C6—C7—C8—C9	56.3 (4)	C23—N7—C24—S3	7.5 (4)
C7—C8—C9—C10	−57.5 (4)	N9—N8—C24—N7	14.1 (4)
N3—C5—C10—C9	131.6 (3)	N9—N8—C24—S3	−169.0 (2)
C6—C5—C10—C9	−51.0 (3)	N8—N9—C25—C30	−179.9 (3)
C8—C9—C10—C5	53.9 (3)	N8—N9—C25—C26	1.2 (4)
C14—N5—N6—C15	174.4 (3)	N9—C25—C26—C27	−131.3 (3)
C14—N4—C13—C12	89.9 (3)	C30—C25—C26—C27	49.8 (4)
C11—C12—C13—N4	129.4 (4)	C25—C26—C27—C28	−52.2 (3)
C13—N4—C14—N5	−179.4 (2)	C26—C27—C28—C29	56.3 (4)
C13—N4—C14—S2	2.0 (4)	C27—C28—C29—C30	−56.2 (4)
N6—N5—C14—N4	7.8 (4)	N9—C25—C30—C29	131.0 (3)
N6—N5—C14—S2	−173.5 (2)	C26—C25—C30—C29	−49.9 (4)
N5—N6—C15—C20	−178.5 (3)	C28—C29—C30—C25	52.5 (4)
N5—N6—C15—C16	0.1 (5)		

### Hydrogen-bond geometry (Å, º)

**Table:**

*D*—H···*A*	*D*—H	H···*A*	*D*···*A*	*D*—H···*A*
N8—H8···S1^i^	0.91	2.47	3.356 (2)	164
N1—H1*A*···N3	0.91	2.14	2.620 (3)	112
N1—H1*A*···N6	0.91	2.40	3.185 (3)	145
N2—H2*A*···S3^ii^	0.91	2.42	3.290 (2)	161
C10—H10*A*···S3^ii^	0.99	2.76	3.516 (3)	133
C10—H10*B*···N9^iii^	0.99	2.67	3.590 (4)	155
N4—H4*A*···N3	0.91	2.15	2.979 (3)	152
N5—H5*A*···N9	0.91	2.24	3.153 (3)	176
C16—H16*B*···N9	0.99	2.42	3.410 (4)	177
N7—H7···S2	0.91	2.51	3.363 (2)	155
N7—H7···N9	0.91	2.25	2.638 (3)	105
C26—H26*A*···N2^iv^	0.99	2.67	3.626 (4)	163
C26—H26*A*···N3^iv^	0.99	2.60	3.499 (4)	152
C26—H26*B*···S1^i^	0.99	2.91	3.619 (3)	129

Symmetry codes: (i) *x*+1, *y*−1, *z*; (ii) *x*−1, *y*+1, *z*; (iii) *x*, *y*+1, *z*; (iv) *x*, *y*−1, *z*.

## References

[bb1] Akkurt, M., Mague, J. T., Mohamed, S. K., Hassan, A. A. & Albayati, M. R. (2014*a*). *Acta Cryst.* E**70**, o478–o479. 10.1107/S1600536814006229 PMC3998596 24826173

[bb2] Akkurt, M., Mohamed, S. K., Mague, J. T., Hassan, A. A. & Albayati, M. R. (2014*b*). *Acta Cryst.* E**70**, o359. 10.1107/S1600536814003948 PMC3998488 24765045

[bb3] Bahadur, S. & Goel, A. K. (1976). *Indian J. Pharm.* **38**, 71–73.

[bb4] Bahat, M. A., Siddiqui, N. & Khan, S. A. (2006). *Indian J. Pharm. Sci.* **68**, 120–124.

[bb5] Brandenburg, K. & Putz, H. (2012). *DIAMOND* Crystal Impact GbR, Bonn, Germany.

[bb6] Bruker (2013). *APEX2*, *SADABS* and *SAINT* Bruker AXS Inc., Madison, Wisconsin, USA.

[bb7] Kalyoncuoğlu, N., Rollas, S., Sür-Altiner, D., Yeğenoğlu, Y. & Anğ, Ö. (1992). *Pharmazie*, **47**, 796–797. 1480660

[bb8] Mague, J. T., Akkurt, M., Mohamed, S. K., Hassan, A. A. & Albayati, M. R. (2014). *Acta Cryst.* E**70**, o366–o367. 10.1107/S1600536814004048 PMC3998438 24765050

[bb9] Mohamed, S. K., Mague, J. T., Akkurt, M., Hassan, A. A. & Albayati, M. R. (2014). *Acta Cryst.* E**70**, o640. 10.1107/S1600536814009817 PMC4051002 24940227

[bb10] Qandil, A. M., Tumah, H. N. & Hassan, M. A. (2006). *Acta Pharm. Sci.* **48**, 95–107.

[bb11] Sheldrick, G. M. (2008). *Acta Cryst.* A**64**, 112–122. 10.1107/S0108767307043930 18156677

[bb12] Singh, N. K., Singh, S. B., Shrivastav, A. & Singh, S. M. (2001). *Proc. Indian Acad. Sci* (*Chem. Sci*), **113**, 257–273.

